# Superresolution based on coherent thermal radiation with selective information

**DOI:** 10.1186/s11671-025-04209-7

**Published:** 2025-02-13

**Authors:** Duan-Hsin Huang, Chih-Wei Chang

**Affiliations:** 1 Center for Condensed Matter Sciences, National Taiwan University, Taipei, 10617 Taiwan; 2 Center of Atomic Initiative for New Materials (AI-MAT), National Taiwan University, Taipei, 10617 Taiwan

**Keywords:** Superresolution, Thermal radiation, Optical microscopy, Phonon-polariton

## Abstract

We reexamine superresolution methods that may have been overlooked by previous optical microscopy techniques. For a one-dimensional (1D) system, we show that maximizing the information capacity of an imaging system is not a necessary condition for surpassing the Abbe diffraction limit. Specifically, the spatial resolution of two coherent emitters can go beyond the Abbe diffraction limit if an appropriate information zone, but not the full information zone, is selected for far-field imaging. Based on this principle, we show that *λ*/2.6 superresolution can be easily achieved for two coherent thermal radiative sources with a sufficiently large phase difference. Similar effects can be found for a 1D array of thermal radiative sources coupled by surface phonon polaritons. Introducing a dielectric microsphere into the system can further enhance the phase difference among the radiative sources, achieving superresolution better than *λ*/4. The concept and method presented here can be implemented to enhance the spatial resolution of thermal imaging.

**Supplementary Information:**

The online version contains supplementary material available at 10.1186/s11671-025-04209-7.

## Introduction

Although an optical image can serve a wide range of applications, a fundamental description of its purpose is to deliver the required information, but not the full information, of an investigated object correctly. On the other hand, because no physical quantity measurement would give infinitely precise results in the presence of noise, an optical system that transmits the required information via light must be limited by bandwidth and noise. Thus, an optic microscope can be regarded as a special instrument resolving the problem of signal recovery. Mathematically, a general case of signal recovery can be expressed as the Fredholm integral equation of the first kind,

1 $${\int }_{a}^{b}K\left(x,y\right){f}_{0}\left(y\right)dy={F}_{0}(x)$$

where *f*_0_(*y*) is the unknown function to be determined, *F*_0_(*x*) is the result of the measurement, and *K*(*x*,*y*) is the point spread function (PSF). However, the general solution of Eq. (1) is difficult to obtain, and various efforts are being devoted to finding special solutions for it [1, 2]. From this point of view, Rayleigh's criteria of the resolution limit of a microscope is an example that provides a simplified solution for Eq. (1) by finding an effective width (Δ) of *K*(*x*,*y*) at the far field ($y \gg \Delta$) to obey

2 $$\left|K\left(x,y\right)/K\left(x,0\right)\right|\gg 1$$

Historically, Shannon formulated the fundamental limit of the maximum information that can be transmitted in a noisy channel without explicitly solving Eq. (1). The application of the Shannon theorem to an optic microscope has been discussed in many works [3–11]. It imposes a fundamental limit of spatial resolution (Δ) [10]:

3 $$\Delta =\frac{\lambda }{2}\frac{1}{{log}_{2}\sqrt{1+2{SNR}_{eff}}}$$

where *SNR*_eff_ is the effective signal-to-noise ratio of the detector, and *λ* is the wavelength of the incident light. Considering a practical detector with *SNR*_eff_ = 1000, Eq. (3) gives Δ < *λ*/10, which is much better than the Abbe's diffraction limit Δ ~ *λ*/2. The result implies that there is still much room for improvement, even though Eq. (1) is difficult to solve. Recently, with the advancement of neural networks, more improvements in Δ can now be achieved even without explicit algorithms [11, 12].

Because of the invariance of information capacity in an imaging system [13–16], a lot of works have been developed to get more information in a desired dimension while compromising unwanted information in another dimension [11]. The far-field superresolution techniques, such as structural illumination [17], super-oscillation lens [18], and hyperlens [19, 20], extend the spatial bandwidth beyond the cut-off frequency of Eq. (2) through advanced fabrication processes to create specialized lenses. In addition, post-image-processing employing assumptions of sparsity or prior knowledge of samples can further aid information retrieval [12, 21, 22]. We also note that although the maximum information capacity of an imaging system sets the ultimate Δ, the superresolution techniques discussed above have implicitly assumed that better Δ can always be achieved when the maximum available information of the system is used. The implicit assumption suggests that whenever the setup is available, a microscope user should always collect as much light as possible at the far field to achieve better spatial resolution.

The implicit assumption may not always be correct when the coherence of light is introduced to an optical system. In this paper, we present a counterexample to the implicit assumption, demonstrating that superresolution can be achieved without utilizing the full information emitted from the sources in a one-dimensional (1D) system. We apply this new method in a 1D system to enhance the spatial resolution beyond the diffraction limit of thermal imaging when the radiative sources are coherent.

## The principle of coherent superresolution with selective information

**Fig. 1:**
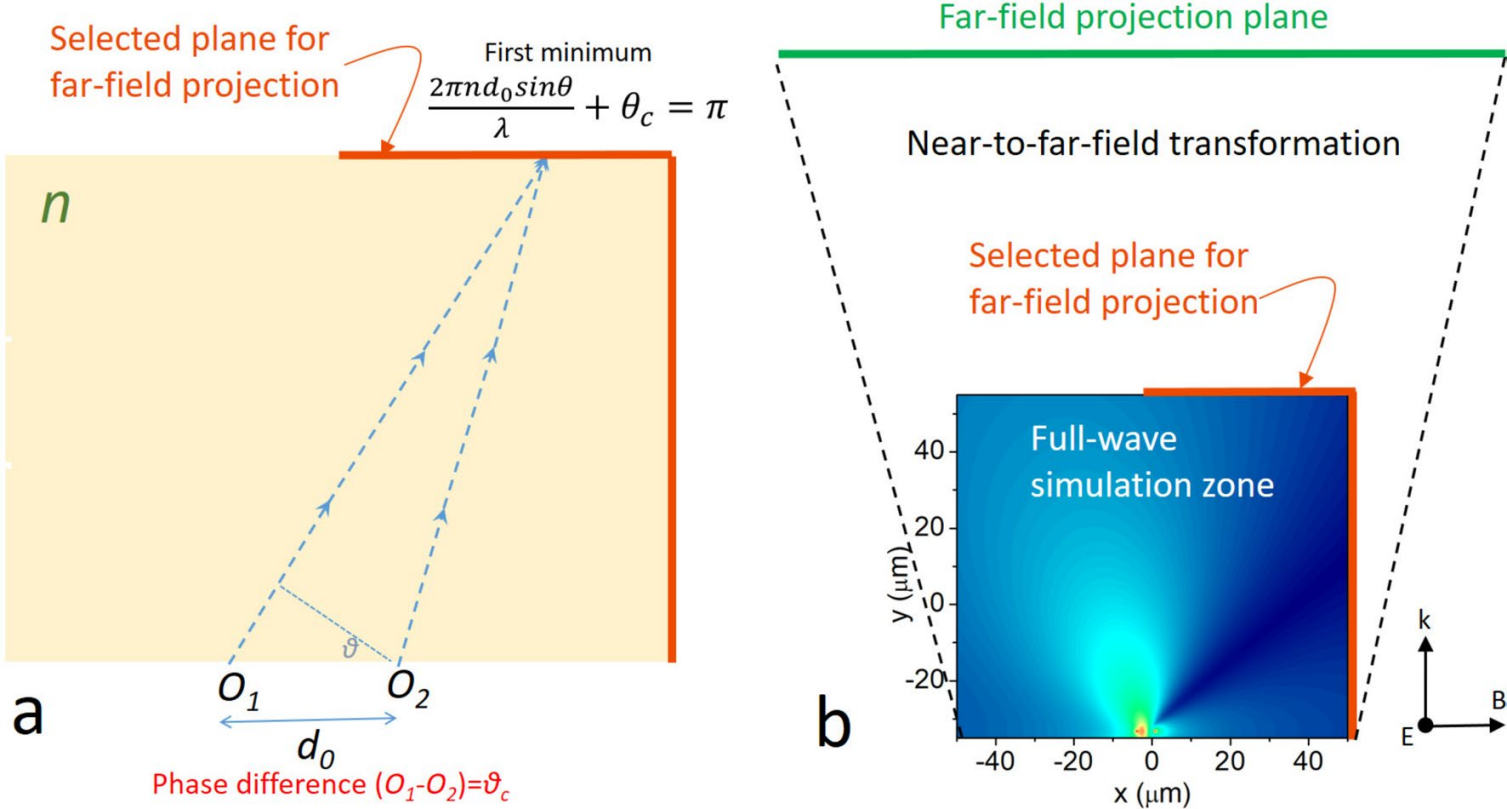
The principle of coherent superresolution with selective information (CSSI). **a** When there is a phase difference (*θ*_*c*_) between two coherent emitters (denoted by *O*_*1*_ and *O*_*2*_, immersed in a media of refractive index *n*), the first minimum of the diffracted light will appear at a smaller angle (expressed by Eqs. (4 and 5)) than that of an ordinary reflective grating (*θ*_*c*_ = 0). Selecting the correct zone for imaging (the orange line) enables a far-field observer to distinguish two point sources beyond the diffraction limit, a key feature of CSSI. **b** The simulation architecture of CSSI. The emitters with polarization perpendicular to plane are located at the bottom of the full-wave simulation zone. To implement CSSI, a selected plane at the boundary of the full-wave simulation zone is chosen for transforming the near-field electromagnetic waves to the far-field. Then the information at the far-field project plane is used for creating virtual image of the objects. In our work, *n* = 1 is set in our simulations

We first reexamine the diffraction limit using a classic example of two coherent point sources in a 1D system (separated by a distance *d*_*0*_, and immersed in a media with refractive index *n*) emitting lights with wavelength *λ* and a phase difference *θ*_*c*_, as shown in Fig. 1. The minimum of the first destructive interference will occur at an angle *θ*, following the relation *2πnd*_*0*_*/λ*sin*θ* = *π*. Similar to the classic example of resolving two incoherent sources by identifying a local intensity minimum at the far-field image, a microscope objective with numerical aperture (*NA* = *n*sin*θ′*, where *θ′* is one-half angular aperture of the objective) is able to distinguish the two emitters only when it can detect the minimum. So, we have sin*θ* = *λ/2nd*_*0*_ < sin*θ′,* and the minimally resolvable distance is *d*_*0*_ = *λ/*2*NA*, which is Abbe's resolution limit for two coherent emitters.

Now, consider that there is a difference between the two coherent emitters. The condition for observing the first destructive interference changes to *2πnd*_*0*_sin*θ/λ* + *θ*_*c*_ = *π*. Correspondently, the criteria for resolving the two emitters is relaxed:

4 $$\frac{\lambda }{2n}\left(\frac{1}{{d}_{0}}-\frac{1}{{\lambda }_{c}}\right)<\text{sin}\theta^{\prime}$$

where *λ*_*c*_ = *2πd*_*0*_*/θ*_*c*_ is a characteristic length. Except for *θ*_*c*_ = 0°, now the condition for destructive interference is relaxed only at one side of the imaging plane but not the other. Thus, only half of the emitted lights are used for imaging, as shown in Fig. 1. Because information capacity increases with numerical aperture, imaging using half of the emitted light indicates a reduced and selective information capacity is used. Thus, we name the technique coherent superresolution with selective information (CSSI), and it is a key factor that has been overlooked by previous works. Compared to Abbe's diffraction limit, CSSI has the minimally resolvable distance (*d*) that obeys:

5 $$\frac{1}{d}=\frac{1}{{d}_{0}}+\frac{1}{{\lambda }_{c}}$$

which shows that *d* is a harmonic mean of *d*_*0*_ and *λ*_*c*_. Thus, *d* is always smaller than *d*_*0*_ or *λ*_*c*_, surpassing the diffraction limit. Note that although it has been known that *θ*_*c*_ = 180° can always have an intensity minimum at the image plane [23], the concept of CSSI has not been discussed to our knowledge. Mathematically, Eq. (5) resembles many other far-field superresolution techniques, for example, two-photon imaging, structural illumination, and hyperlens. In the following, we will discuss their differences and show that Eq. (5) is a new concept of optic imaging technique.

## Results and discussion

### Comparing CSSI with other superresolution techniques

Two-photon imaging is based on the nonlinear optic phenomena that two incoming coherent lasers are up-converted by a target object and emit at higher frequencies. Thus, the outgoing light would have shorter wavelengths and better spatial resolution. The mathematical basis of the two-photon imaging is *k*_*1*_ + *k*_*2*_ = *k*_*3*_ and *ω*_*1*_ + *ω*_*2*_ = *ω*_*3*_ (where *k*_*i*_ is the wave factor and *ω*_*i*_ is the frequency of the light). Although two-photon imaging is advantageous in detecting tissue structures 1 mm below the surface [24], its requirements of high-intensity lasers to excite the target sample have been the major drawback of its applications [25].

Structural illumination employs a shift of the frequency spectrum of a sample by illuminating it using a periodic structure with a spatial modulation frequency *k*_*mod*_ [17]. The superimposed image would have Moiré fringes that contain information about high-frequency patterns that would otherwise not be visible. Mathematically, its principle can be expressed as *k*_*in*_ + *k*_*mod*_ = *k*_*out*_, (*k*_*mod*_ < *k*_*in*_). It is often used for fluorescent imaging of biological samples [26]. However, because of the need to capture 360° frequency domain, rotating the modulated pattern and computational post-processing are required [26]. Furthermore, because of the fundamental constraint *k*_*mod*_ < *k*_*in*_, its spatial resolution cannot go beyond 2*k*_*in*_.

Hyperlens employs a sophisticated lens design that modifies its frequency contour at the Fourier space into a hyperbolic curve, extending the spatial frequency in the radial direction while compressing the azimuthal information [19, 20]. Its mathematical basis is *k*_*in*_ + *k*_*mod*_ = *k*_*out*_ without the additional constraint of *k*_*mod*_ < *k*_*in*_ mentioned in structural illumination [27]. Although the concept has been benefitted by the introduction of metamaterials, the fabrication difficulties have limited its applications [11].

Thus, despite the similarities in the mathematical formulation, we now understand the fundamental difference between Eq. (5) and other techniques. Unlike the two-photon imaging, CSSI does not require optical nonlinearity. Unlike structural illumination or hyperlens, the phase difference required by CSSI does not necessarily demand modulated structures. In fact, coherent light sources and selecting an appropriate zone for imaging are two important features of our technique. Although the former has been extensively investigated in literature, the latter has never been discussed to our knowledge.

### Simulation of CSSI from two emitters

Although thermal radiative light sources such as incandescent light bulbs are often considered to be incoherent, employing surface phonon-polaritons as a coupling mediator to realize coherent thermal radiation has been experimentally demonstrated in various polar materials with engineered structures [28–39]. In general, artificially designed meta-surfaces can be used for controlling the dispersion relation of surface phonon-polaritons as well as the *θ*_*c*_ [40]. Thus, coherent thermal radiation is an ideal platform to explore our ideas. We have employed FDTD simulation using *Lumerical-FDTD* to test the concept of CSSI. We put point sources with a separation *d*_*0*_ and impose a phase difference *θ*_*c*_. As shown in Fig. 1b, a selected plane at the boundary of the full-wave simulation zone is used to transform the near-field electromagnetic waves into forward-propagating waves to the far-field [41]. The magnitude and phase of the angular spectrum at the far-field projection are then Fourier-transformed and backward propagated to get the intensity profile of the virtual image of the object at the far field [41]. After backward propagation, we adjust the position of the image plane to get a focused virtual image. The process will naturally give a magnified virtual image at the far field [41]. The above procedures are conducted using *Lumerical-FDTD* and have been validated experimentally [41]. Controlled simulations of incoherent imaging for two-point emitters are carried out using *θ*_*c*_ = 90°.

**Fig. 2:**
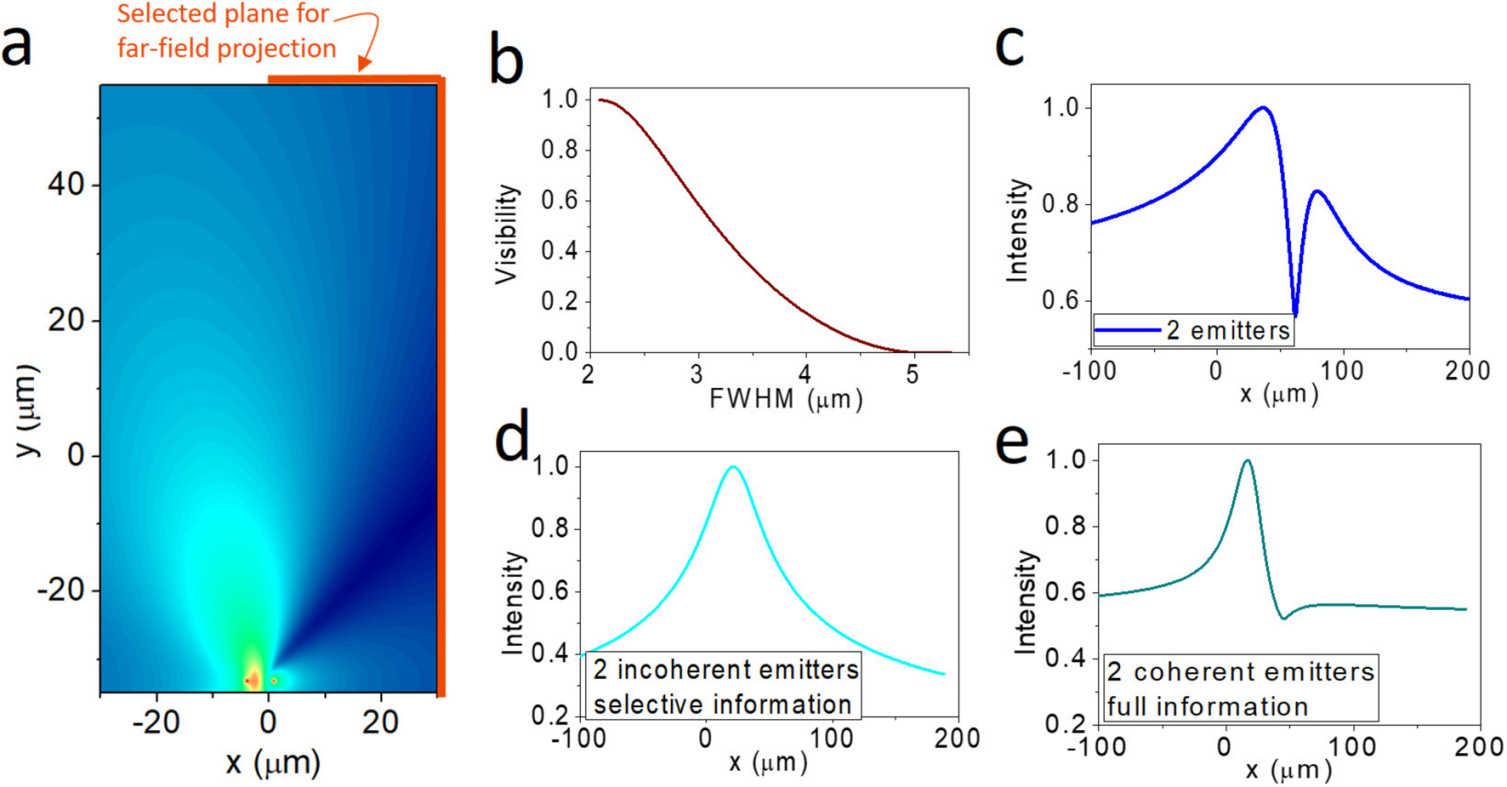
CSSI of two coherent emitters. **a** Simulated intensity profile of two point sources separated by *d*_0_ = 4.7 µm and emitting *λ* = 10 µm. The selected plane for far-field projection is denoted as the orange line. **b** The relation between FWHM and visibility. The maximum visibility read from the far-field intensity profile is used for quantifying the FWHM. **c** The far-field (virtual image) intensity profile of two coherent emitters with *θ*_*c*_ = 80°. Here, the visibility is found to be 0.186, corresponding to *λ/*FWHM = 2.56. **d** The intensity profile of the virtual image for two incoherent emitters and imaging with selected information. **e** The intensity profile of the virtual image for two coherent emitters with *θ*_*c*_ = 80° using full information

We have numerically simulated the system shown in Fig. 2a using two-point sources with *d*_0_ = 4.7 µm and *λ* = 10 µm. To quantify the resolution, we first define the visibility = (*I*_*max*_ − *I*_*min*_)/(*I*_*max*_ + *I*_*min*_) (where *I*_*max*_ and *I*_*min*_ are, respectively, the local intensity maximum and minimum of the far-field intensity profile; see supplementary information S1). To quantify the spatial resolution, we first establish the relation between the visibility and the full-width-at-half-maximum (FWHM) of two incoherent emitters and then use it to determine the effective FWHM of the images obtained by CSSI (see supplementary information S1). Figure 2b shows the relation between FWHM and visibility obtained from the deconvolution of various emission *λ*'s with a fixed *d*_0_ = 4.7 µm. We find that the visibility vanishes when FWHM = 4.83 µm = 1.03*d*_0_. In the following, we will find that the effective FWHMs obtained at different conditions can be regarded as *d* in Eq. (5). We choose the highest visibility in the far-field intensity profile to quantify the resolution. When *θ*_*c*_ = 80°, the far-field intensity profile at the selected imaging zone is shown in Fig. 2c. It displays two intensity peaks, correctly suggesting the presence of two emitters at the source. We also find that the two peaks show unequal intensities even though the magnitude of the two emitters is set to be equal. It is a common feature of CSSI, and it originates from the *θ*_*c*_ and the selected zone of imaging that causes the asymmetric intensity profile. From Fig. 2b, we find FWHM < *λ*/2.6 (or FWHM = 0.82*d*_0_) when *θ*_*c*_ = 80°, surpassing the diffraction limit.

To prove that the coherence of the emitters and the selected information are the two key factors of CSSI, we provide two controlled simulations, one for incoherent imaging and another for coherent imaging with full information capacity, as shown in Fig. 2d, e (see supplementary information S2). Compared with Fig. 2c, we can see that neither incoherent imaging nor coherent imaging using full zone can clearly resolve the two-point sources at the far field.

**Fig. 3:**
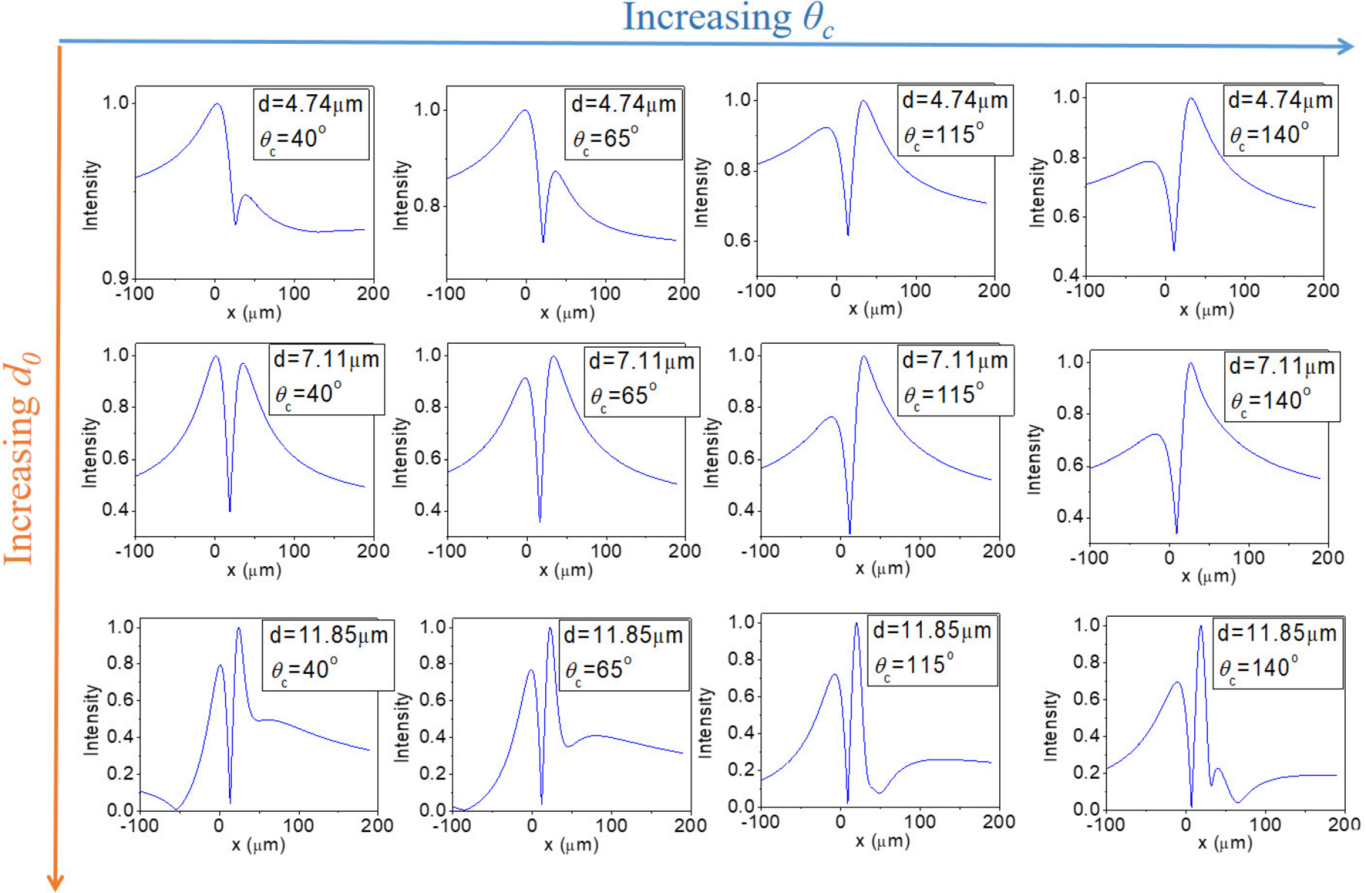
Simulated far-field (virtual image) intensity profile of two point sources emitting *λ* = 10 µm for various *d*_*0*_'s and *θ*_*c*_'s. Note that image speckles would appear once *d*_*0*_ or *θ*_*c*_ becomes too large

We have conducted a series of simulations for various *d*_*0*_ or *θ*_*c*_. As shown in Fig. 3, we find that CSSI can easily go beyond the diffraction limit and correctly resolve the two emitters. Besides, increasing *d*_*0*_ or *θ*_*c*_ would generally improve visibility. These features can be understood from Eq. (5) that the resolving power increases as *θ*_*c*_ increases. Furthermore, intensity asymmetry is a common phenomenon for CSSI. Lastly, when *d*_*0*_ or *θ*_*c*_ becomes too large, image speckles could appear once the condition for the second destructive interference is satisfied (i.e., *2πnd*sin*θ/λ* + *θ*_*c*_ = *3π*).

### Simulation of CSSI from five emitters

**Fig. 4:**
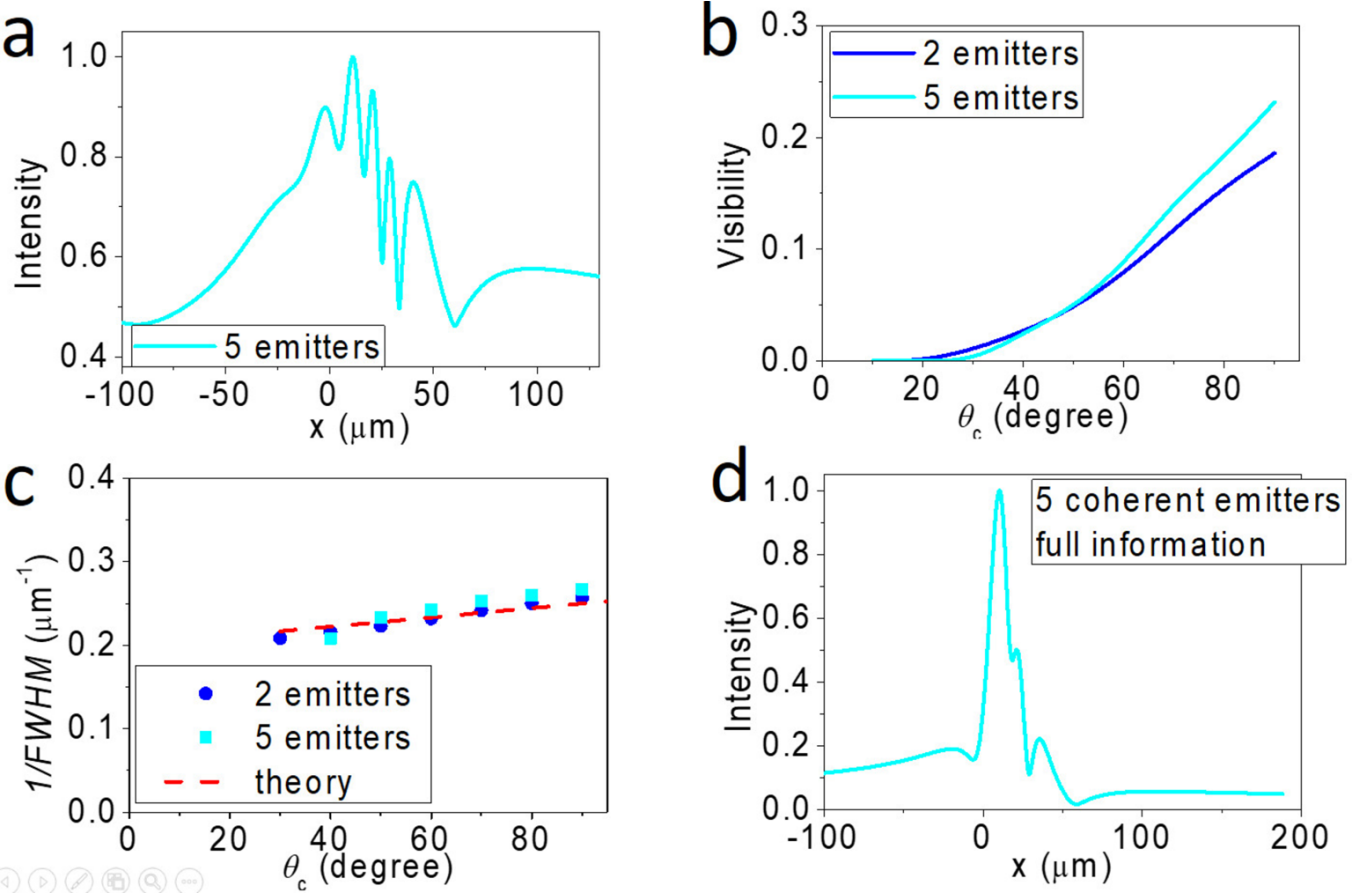
Simulated results of five point sources separated by *d*_0_ = 4.7 µm and emitting *λ* = 10 µm. **a** Simulated far-field (virtual image) intensity profile of five point sources with nearest neighbor *θ*_*c*_(*O*_*1*_–*O*_*2*_) = 80°. Here, the highest visibility is found to be 0.232, corresponding to *λ/*FWHM = 2.5. **b** Far-field (virtual image) visibility vs. *θ*_*c*_ for two- (blue curve) and five- (cyan curve) point emitters. **c** The corresponding 1/FWHM vs. *θ*_*c*_ for the data shown in **b**. The theoretical prediction of Eq. (5) is shown as the red dashed line. **d** The virtual image of controlled simulation of five coherent emitters (*θ*_*c*_ = 80°) using full information

The above statements still hold for an array of point emitters with nearest neighbor *θ*_*c*_ = 80°. As demonstrated in Fig. 4a, five intensity peaks are observed, in agreement with the number of source emitters. We find that the visibility improves with increasing *θ*_*c*_*,* and the trend is nearly independent of the number of emitters, as shown in Fig. 4b. In general, the far-field profile can correctly resolve the number of point sources even though the intensity distributions may be inhomogeneous and the FWHM may be uneven. If choosing the maximum visibility in an intensity profile to represent the best FWHM, we find our simulations quantitatively agree with Eq. (5), as shown in Fig. 4c. On the other hand, controlled simulations using a full zone always fail to resolve the number of emitters, as shown in Fig. 4d.

### Simulation of CSSI from coherent emitters underneath a lens

We now discuss whether it is possible to enhance the spatial resolution further. Since the discovery of superresolution assisted by dielectric microlenses [42, 43], it has been shown that microlenses exhibiting diameters less than ~ 140*λ* would exhibit properties such as resolving power or magnification deviating from those of ordinary solid immersion lenses (with diameters larger than 200*λ*) [43–49]. The deviation becomes more pronounced when their diameters are less than 20*λ*. Many mechanisms have been put forward to explain the observed superresolution [43, 50–54], but there are still some fundamental disagreements. For example, a sharply focused spot (dubbed "photonic nanojet") formed at the focal point of a microlens has been frequently suggested to be the mechanism for the superresolution [43–47]. However, numerical simulations and experimental studies found the width of the nanojet to be ~ *λ*/2*,* disagreeing with many observations [48, 49]. Later, it was suggested that a resonant nanojet with a narrower width (*λ*/3–*λ*/4) would appear once the incident light satisfies the condition of whisper gallery mode of a microsphere [51, 55, 56]. However, the resonant condition is difficult to meet, and it can be easily destroyed once the geometry of the lens deviates from a perfect sphere. In fact, none of the previous simulations can reproduce resolution better than *λ*/4, disagreeing with many experimental results.

**Fig. 5:**
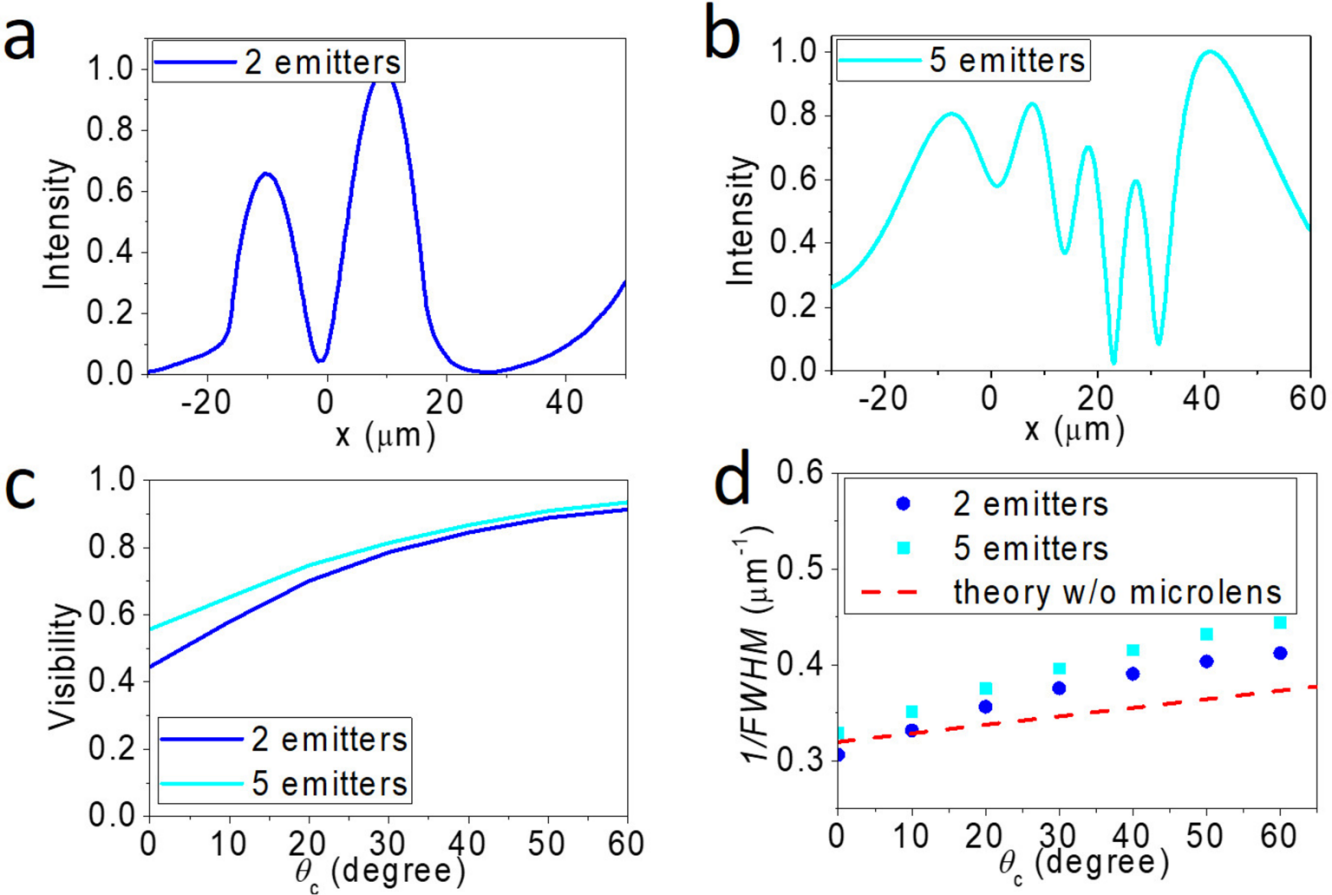
Simulated far-field (virtual image) intensity profile of coherent emitters with *θ*_*c*_(*O*_*1*_–*O*_*2*_) = 60°, separated by *d*_0_ = 4.7 µm, and emitting *λ* = 10 µm under a dielectric lens with n = 1.5 and diameter = 63 µm. **a** Simulated far-field (virtual image) intensity profile of two emitters. Here, the visibility is found to be 0.91, corresponding to *λ/*FWHM = 4.13. **b** Simulated far-field (virtual image) intensity profile of five emitters. Here, the highest visibility is found to be 0.93, corresponding to *λ/*FWHM = 4.15. **c** Far-field visibility vs. *θ*_*c*_ for two- (blue curve) and five- (cyan curve) point emitters under a n = 1.5 dielectric lens. **d** The corresponding 1/FWHM vs. *θ*_*c*_ for the data shown in **c**. The theoretical prediction of Eq. (5) (using *NA* = 1.5) is shown as the red dashed line

Inspired by these works, we employ a dielectric lens for imaging coherent thermal radiation under a selected zone. Figure 5a illustrates the intensity profile of two coherent thermal radiators emitting *λ* = 10 µm with *θ*_*c*_ = 60°. The two emitters are separated by *d*_0_ = 4.74 µm, located underneath a 63 µm of diameter microlens with refractive index *n* = 1.5. Comparing with Fig. 2a, we find that it resolves the two emitters with better contrast even though the *θ*_*c*_ is smaller here. As shown in Fig. 5b, we have also simulated the results of five coherent thermal radiators emitting *λ* = 10 µm with *θ*_*c*_ = 60°. It clearly resolves the five emitters with an inhomogeneous intensity distribution. Similar to Fig. 4b, Fig. 5c shows the visibility increases with increasing *θ*_*c*_. Here, the highest visibility is found to be 0.93, corresponding to *λ/*FWHM = 4.15.

To investigate the effect of the microlens, we plot the simulated data into 1/FWHM vs. *θ*_*c*_ and compare them with the prediction of Eq. (5). From Fig. 5d, we find that the presence of a microlens makes 1/FWHM versus *θ*_*c*_ relation deviate from Eq. (5) and the 1/FWHM increases with a higher slope than the prediction of Eq. (5). Besides, the slope is steeper for the five-point emitters than that for the two-point emitters. Because of the linear relation shown in Fig. 5d, we empirically introduce an enhanced factor *f* to account for the effective phase difference (*fθ*_*c*_) observed in the microlens. Apparently, *f* would be a complex function of the number of emitters, their locations, the *n* of the microsphere, and the diameter of the microsphere. Although investigating the physics behind *f* is beyond the scope of the paper, we have learned that the overall effect of introducing the microsphere can be simplified to a single parameter *f*.

We now discuss possible scenarios that can implement CSSI. The *θ*_*c*_ of coherent thermal radiation can be created by applying a temperature gradient along a polar material, in which an added heat flux has been experimentally observed [57]. Alternatively, CSSI can be used for imaging samples deposited on a metasurface with surface plasmons or surface phonon-polaritons excited by lasers or other external light sources [58–61]. In general, *θ*_*c*_ can always be created once there are interactions between two sources. On the other hand, because selecting the correct information zone will give better resolution than the other in 1D cases, one can easily carry out CSSI by tilting/rotating the sample, changing the direction of the temperature gradient, or varying the incident angle of an external light source. Because the concept introduced here is simple and does not incorporate any quantum [62–66] or nonlinear effects [67, 68], we anticipate that the technique can be extended to 2D imaging. For example, similar to the methods used in structural illumination, CSSI in 2D can be carried out by rotating the sample or the external light source. Image speckles can be removed by using an algorithm that compares CSSI with images from incoherent light sources or employs full information.

## Conclusion

In conclusion, existing superresolution techniques generally assume that higher spatial resolution can be achieved by utilizing the maximum information received in the far field. Here, we provide a counterexample demonstrating that spatial resolution going beyond the Abbe diffraction limit can be obtained in the far field when sources emit coherent light and only selective information is used for imaging. This concept may be realized using coherent thermal radiation mediated by surface phonon polaritons. Additionally, we show that spatial resolution can be further enhanced by introducing a dielectric microsphere to the system. Thus, beyond thermal imaging, this new technique may inspire advancements in infrared microscopy, such as Fourier transform infrared spectroscopy.

## Supplementary Information

Additional file 1.

## Data Availability

The data supporting the findings of this study are available within the paper and its supplementary information file.
