# Supplementary material for: Superresolution based on coherent thermal radiation with selective information
Source: Discov Nano. 2025 Feb 13;20(1):34. doi: 10.1186/s11671-025-04209-7 (PMC11825967; doi:10.1186/s11671-025-04209-7)
Supplement: Supplementary file 1 — Additional file 1. [file 11671_2025_4209_MOESM1_ESM.docx]

**Supplementary information for**

**Superresolution based on coherent thermal radiation with selective information**

*Duan-Hsin Huang,^1^ and Chih-Wei Chang^1,2^*

^1^Center for Condensed Matter Sciences, National Taiwan University, Taipei 10617, Taiwan

^2^Center of Atomic Initiative for New Materials (AI-MAT), National Taiwan University, Taipei, 10617, Taiwan

**S1. Visibility vs FWHM.** Figure S1 shows a representative intensity profile at the image plane at the far field. For each local intensity maximum and minimum, we can calculate its visibility = (*I_max_*−*I_min_*)/(*I_max_*+*I_min_*). The maximum visibility is chosen to calculate the FWHM in Fig. S1 and in the main text.


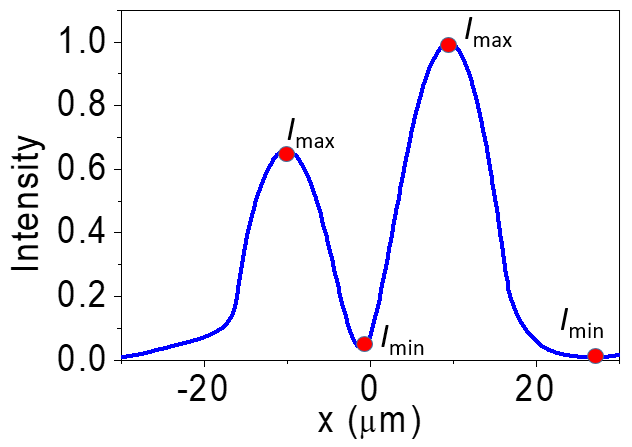


**Figure S1.** A representative intensity profile of the image plane at the far field. The local intensity maximums and minimums are denoted as red circles. Maximum visibility is chosen to plot the relation between FWMH and visibility in the main text figures.

The deconvolution method is obtained by reading the maximum visibility of the far-field intensity and using Fig. 2(b) in the main text to obtain the FWHM. The relation between visibility and FWHM is established using the procedures described in Fig. S2. First, the FWHM of a point source emitting a wavelength (λ) is directly obtained from its far-field intensity profile. Then, another point source emitting identical λ but separated from the first point source by *d*_0_=4.7 µm and *θ_c_*=90° is added to the simulation. From the far-field intensity profile shown in Fig. S2(b), we can obtain its visibility. Because the capability of identifying an intensity minimum is usually used as Abbe’s (and also Rayleigh’s) criteria for defining the resolution limit for two-point emitters, the visibility or the FWHM can be used for quantification of an optic system. Lastly, the above procedures are repeated for different λ’s and the relation between FWHM and visibility is established, as shown in Fig. S2(c).


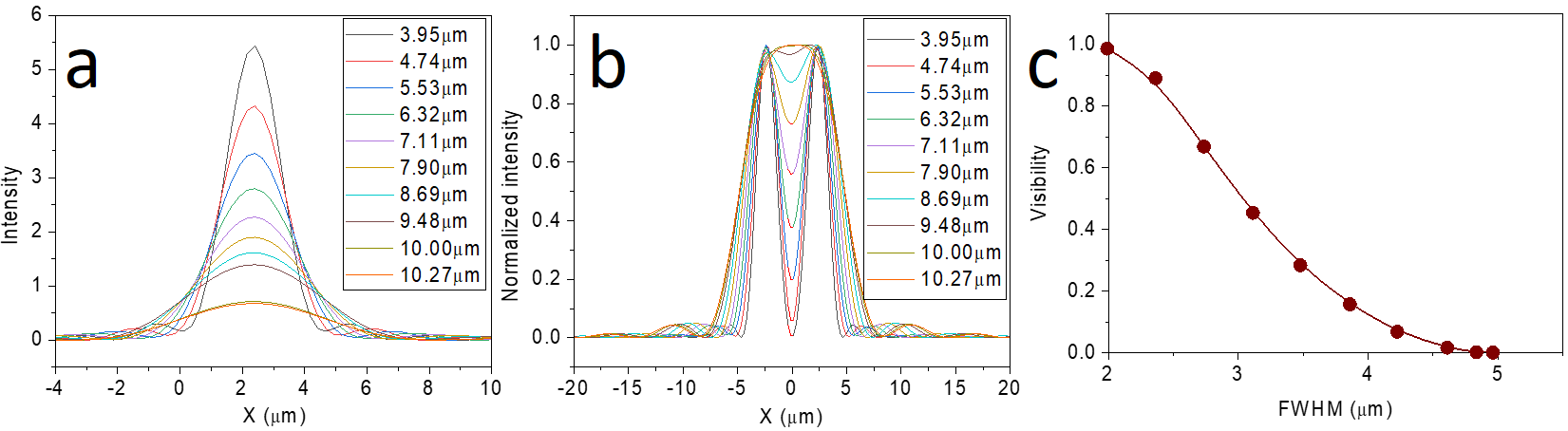


**Figure S2.** The relation between FWHM and visibility. (a) The simulated far-field intensity profile of a point source emitting different λ’s. The FWHM is directly read from each profile. (b) The far-field intensity profile of two incoherent sources emitting different λ’s. Here, the visibility can be directly determined using the method shown in Fig. S1. (c) The established relation between FWHM and visibility, where the data points are from (a) & (b) and the brown curve is from Fig. 2(b).

**S2. Controlled simulations: incoherent imaging and imaging with full information.** To prove that coherent imaging and selective information at the image plane are the two key factors of CSSI, we provide controlled simulations for incoherent imaging and coherent imaging with full information capacity, respectively shown in Figs. S3(a & b).


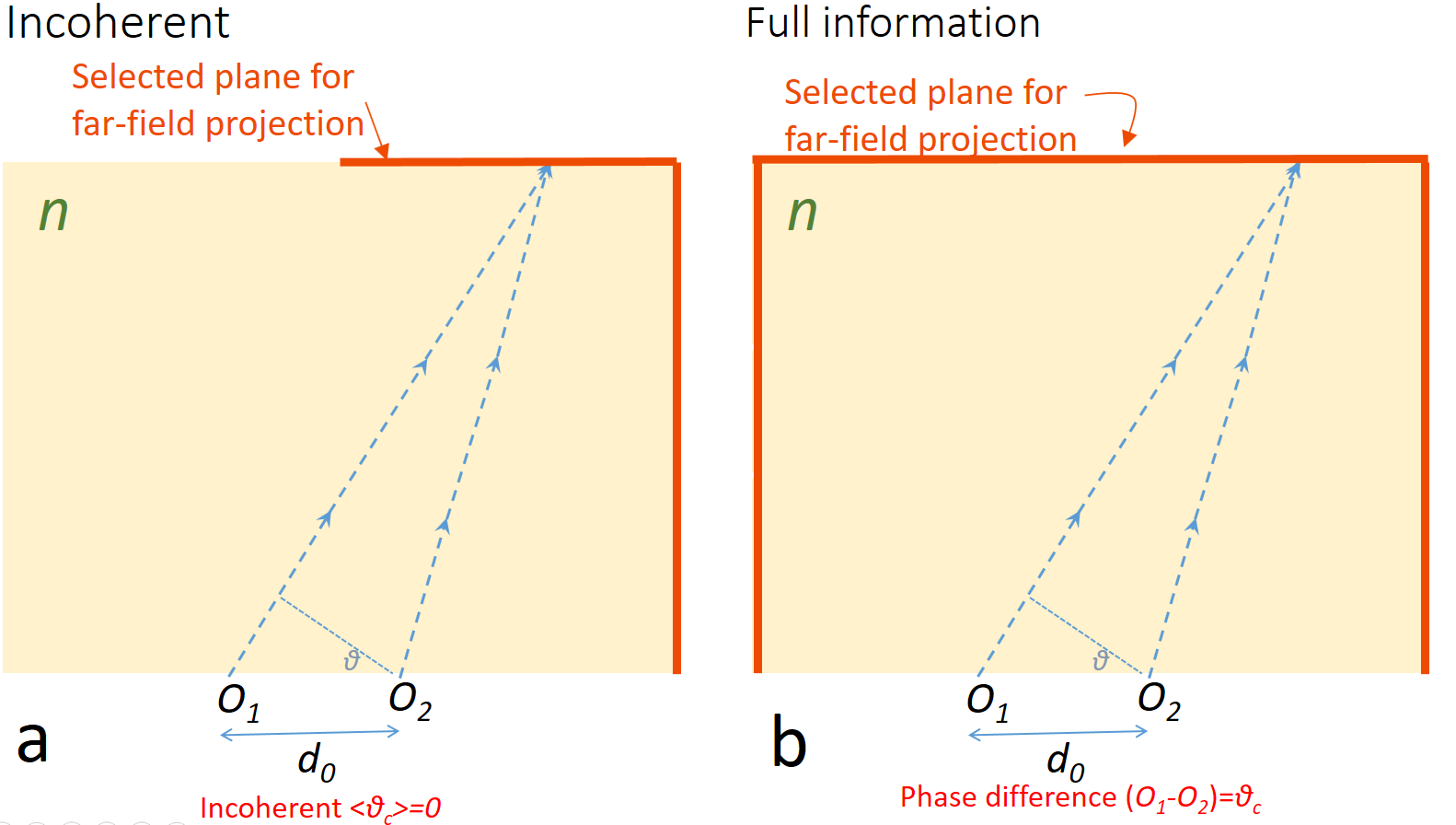


**Figure S3.** Schematic illustrations of two point sources (denoted by *O_1_* and *O_2_*, immersed in a media of refractive index *n*) that are imaged using (a) incoherent imaging with selective information and (b) coherent imaging with full information.
